# Avenciguat: a novel soluble guanylate cyclase activator that affects multiple cell types to inhibit IFN-1 signalling and fibrosis

**DOI:** 10.1093/rheumatology/keaf109

**Published:** 2025-02-22

**Authors:** Julia Kaufman, Gerald Nabozny, Cuong Tran-Manh, Christoph Liebel, Xiang Zhou, Lee-Anne Daley, Chao-Ting Wang, David L Ebenezer, Denis Delic, Christian T Wohnhaas, Thuong Trinh-Minh, Jörg H W Distler

**Affiliations:** Immunology and Respiratory Diseases Research, Boehringer Ingelheim Pharmaceuticals Inc., Ridgefield, CT, USA; Immunology and Respiratory Diseases Research, Boehringer Ingelheim Pharmaceuticals Inc., Ridgefield, CT, USA; Department of Rheumatology, University Hospital Düsseldorf, Heinrich-Heine University Düsseldorf, Düsseldorf, Germany; Hiller Research Center, University Hospital Düsseldorf, Heinrich-Heine University Düsseldorf, Düsseldorf, Germany; Department of Rheumatology, University Hospital Düsseldorf, Heinrich-Heine University Düsseldorf, Düsseldorf, Germany; Hiller Research Center, University Hospital Düsseldorf, Heinrich-Heine University Düsseldorf, Düsseldorf, Germany; Department of Rheumatology, University Hospital Düsseldorf, Heinrich-Heine University Düsseldorf, Düsseldorf, Germany; Hiller Research Center, University Hospital Düsseldorf, Heinrich-Heine University Düsseldorf, Düsseldorf, Germany; State Key Laboratory of Genetic Engineering, School of Life Sciences and Human Phenome Institute, Fudan University, Shanghai, China; Immunology and Respiratory Diseases Research, Boehringer Ingelheim Pharmaceuticals Inc., Ridgefield, CT, USA; Immunology and Respiratory Diseases Research, Boehringer Ingelheim Pharmaceuticals Inc., Ridgefield, CT, USA; Immunology and Respiratory Diseases Research, Boehringer Ingelheim Pharmaceuticals Inc., Ridgefield, CT, USA; Translational Medicine and Clinical Pharmacology, Boehringer Ingelheim Pharma GmbH & Co. KG, Biberach, Germany; Translational Medicine and Clinical Pharmacology, Boehringer Ingelheim Pharma GmbH & Co. KG, Biberach, Germany; Department of Rheumatology, University Hospital Düsseldorf, Heinrich-Heine University Düsseldorf, Düsseldorf, Germany; Hiller Research Center, University Hospital Düsseldorf, Heinrich-Heine University Düsseldorf, Düsseldorf, Germany; Department of Rheumatology, University Hospital Düsseldorf, Heinrich-Heine University Düsseldorf, Düsseldorf, Germany; Hiller Research Center, University Hospital Düsseldorf, Heinrich-Heine University Düsseldorf, Düsseldorf, Germany

**Keywords:** systemic sclerosis, microvasculopathy, fibrosis, immune dysregulation, avenciguat, inflammation, interferon

## Abstract

**Objectives:**

The soluble guanylate cyclase (sGC) stimulator riociguat is approved for the treatment of pulmonary arterial hypertension and may have antifibrotic effects. However, in fibrotic tissues, oxidative stress and hypoxia can render sGC insensitive to sGC stimulators. sGC activators overcome this limitation. Here, we characterize the novel sGC activator, avenciguat, in preclinical models of SSc.

**Methods:**

Human microvascular endothelial cells-dermal (HMVEC-d) cultured in hypoxic conditions and activated human platelet-rich plasma were incubated with varying doses of avenciguat, and the levels of TGF-β2 and human CXC chemokine family ligand 4 (CXCL4) were measured, respectively. Treatment with avenciguat was analysed in mice with bleomycin-induced dermal and pulmonary fibrosis.

**Results:**

Avenciguat reduced hypoxia-induced synthesis of TGF-β2 by HMVEC-d and inhibited CXCL4 release by platelets. Moreover, avenciguat demonstrated antifibrotic effects on bleomycin-induced dermal and pulmonary fibrosis. RNA sequencing of affected skin uncovered a unique profile distinguishing avenciguat from riociguat. Avenciguat treatment resulted in deeper regulation of IFN-1 signalling and genes associated with immune response *vs* riociguat treatment.

**Conclusion:**

In preclinical studies, avenciguat shows the potential to influence vascular, fibrotic and immune-related processes in murine models of SSc. These studies suggest that it may offer therapeutic benefit across multiple aspects of SSc pathophysiology and support the rationale for further investigation in a Phase II clinical trial (VITALISScE™; NCT05559580) of avenciguat in SSc.

Rheumatology key messages

- Current therapies mainly target only one aspect of SSc (microvasculopathy, immune dysregulation or fibrosis).
- Avenciguat, a novel sGC activator, targets the underlying pathophysiology of SSc, even under hypoxic conditions.
- The Phase II trial, VITALISScE™, is assessing the safety/efficacy of avenciguat in patients with SSc.

## Introduction

SSc is an autoimmune CTD characterized by microvascular abnormalities, immune dysregulation and chronic inflammation, preceding progressive skin and organ fibrosis [1, 2]. Endothelial cell apoptosis and subsequent vascular injury lead to hypoxia and oxidative stress [2]. This is associated with overproduction of nitric oxide (NO) and reactive oxygen species, which amplifies endothelial damage and promotes fibrosis, in part via induction of TGF-β [2]. Platelet activation also contributes to the proinflammatory and profibrotic environment [3]. Extracellular matrix accumulation further impairs perfusion and oxygen diffusion, leading to a feed-forward pathogenic loop driving progressive fibrosis [2]. In addition, many patients with SSc have increased expression of IFN-1-regulated genes, including CXC chemokine family ligand 4 (CXCL4), with the level of IFN activation correlating with the severity of organ involvement [4, 5]. Increased CXCL4 expression further contributes to fibrosis progression [6].

The soluble guanylate cyclase (sGC)–cyclic guanosine monophosphate (cGMP) pathway has also been implicated in SSc pathogenesis [7]. Under homeostatic conditions, NO binds to a prosthetic haem group on sGC, permitting cGMP production [8]. Oxidative stress, which is present in patients with SSc, leads to the production of the oxidized, dysfunctional, haem-free form of sGC that is unresponsive to NO. This can lead to a reduction in sGC activity and cGMP production, which in turn impairs downstream cGMP-regulated processes. There are two different types of molecules that target sGC. The sGC stimulators require haem-bound sGC and enhance sGC sensitivity to NO, whereas sGC activators, such as avenciguat, bind directly to haem-free sGC and can activate the enzyme independently of NO [8].

Riociguat, an sGC stimulator, reduces dermal fibrosis in preclinical models of SSc [7] and is approved to treat pulmonary arterial hypertension [9]. In the Phase II RISE-SSc study, though the primary endpoint [change from baseline in modified Rodnan skin score (mRSS) at 52 weeks] was not met, other endpoints showed potential efficacy signals [10].

sGC activators, such as avenciguat, offer conceptual benefits over sGC stimulators [8]. Under hypoxic conditions or oxidative stress, e.g. in SSc skin and other fibrotic tissues, sGC activators bind directly to oxidized haem-free sGC, stabilizing sGC in an active form, leading to stimulation of cGMP production [8].

Several candidates under investigation modulate inflammatory pathways or fibroblast activation [11], but there are currently no approved treatments targeting the interplay between microvascular disease, autoimmunity and fibrosis. Addressing all three aspects may offer the potential for true disease modification of SSc.

Here, we present the first evidence that therapeutic up-regulation of endogenous levels of cGMP through sGC activation may improve vasculopathy and fibrosis as well as regulating IFN responses in SSc.

## Methods

### HMVEC-d hypoxia assay

Human microvascular endothelial cells-dermal (HMVEC-d) (Lonza, Walkersville, MD, USA) were cultured in endothelial basal medium-2 (Lonza, Walkersville, MD, USA) supplemented with endothelial growth medium-2MV (Lonza, Walkersville, MD, USA), 10% fetal bovine serum, 1% glutamax (Gibco, Brooklyn, NY, USA) and 1% penicillin/streptomycin (Gibco, Brooklyn, NY, USA) at 37°C/5% CO_2_. After overnight incubation, cells were transferred to endothelial basal medium-2 supplemented with 2% fetal bovine serum for serum starvation. After 24 h in serum-starved media, cells were treated with avenciguat and incubated in normoxic or hypoxic (1% O_2_) conditions. After 48 h, supernatant was collected and TGF-β2 levels were determined using the U-plex human TGF-β2 assay kit (Meso Scale Discovery, Rockville, MD, USA).

All studies utilizing human cells were performed in accordance with the Human Biospecimen policies of Boehringer Ingelheim.

### CXCL4 release in human PRP

Human whole blood (≤35 ml) was collected and centrifuged at 200 × *g* for 16 min at room temperature. The platelet-rich plasma (PRP) was transferred to a new tube and incubated at room temperature for 5–15 min. Up to 80 µl of PRP was transferred to NUNC™ 1 ml-deep well plates (Thermo Scientific, Waltham, MA, USA) containing up to 560 µl of HEPES-Tyrode BSA buffer and incubated with avenciguat, riociguat, nintedanib or MMF for 30 min at 37°C. Up to 80 µl of 100 µM adenosine 5′-diphosphate (ADP) was added and incubated for 5 min. After incubation, 300 µl/well of stimulated PRP was transferred to sterile 0.22 µm polyvinylidene fluoride 96-well filter plates (Millipore-Sigma, Burlington, MA, USA) and levels of human CXCL4 in the flow-through supernatant were measured using the CXCL4 Quantikine Enzyme Linked Immunosorbent Assay kit (R&D systems, Minneapolis, MN, USA).

### Bleomycin-induced skin and lung fibrosis mouse models

Adult female C57Bl/6 mice (Janvier Labs, Le Genest-Saint-Isle, France) received an s.c. injection of bleomycin (100 μl, 2.5 mg/ml) as described previously [12]. Twice-daily treatment with avenciguat, riociguat (Biomol, Hamburg, Germany) or nintedanib (Boehringer Ingelheim, Biberach an der Riß, Germany) was initiated at week 4. Non-fibrotic controls were injected with 0.9% sodium chloride (NaCl). Treatment groups assessed were: NaCl, bleomycin, nintedanib (60 mg/kg), avenciguat (1, 3 and 10 mg/kg) and riociguat (1 mg/kg) (*n* = 8 per group). Treatments were given orally twice daily, except for nintedanib, which was once daily. After 6 weeks of treatment, skin samples were collected and analysed for markers of fibrosis. Dermal thickness was measured as the distance between the epidermal–dermal border to the dermal–subcutaneous border in arbitrary units from four different sections of the upper back, with two measurements per section, performed in a blinded manner [12]. Myofibroblasts were detected by immunochemistry staining of paraffin-embedded slides and quantified by a blinded reviewer [12]. Collagen was determined via the hydroxyproline assay as described previously [12].

Lung fibrosis was induced via a single intratracheal injection of bleomycin (50 µl, 2.5 mg/ml) at day 0 [13]. Non-fibrotic controls were injected with 0.9% NaCl. Treatment started at day 15 post-administration of bleomycin (*n* = 8 per treatment group). Lung fibrosis was analysed at day 28 post-treatment initiation. Histological evaluation of pulmonary fibrosis was quantified by Ashcroft Scoring [13], performed in a blinded manner. Whole lung sections were stained with Sirius Red (Sigma-Aldrich, St Louis, MO, USA) and total fibrotic area was determined as the percentage of total area covered by Sirius Red [13]. Collagen was determined via the hydroxyproline assay as described previously [13].

RNA was extracted from bleomycin-treated mice following avenciguat and riociguat treatment using the RNeasy Fibrous Tissue Mini Kit (Qiagen, Hilden, Germany) and changes in gene expression were assessed (see Supplementary Material, available at *Rheumatology* online, for further details).

All animal experiments were approved by the ethical committee of the Fudan University Shanghai, China.

## Results

### Avenciguat inhibits hypoxia-induced TGF-β2 production

**Figure 1.**
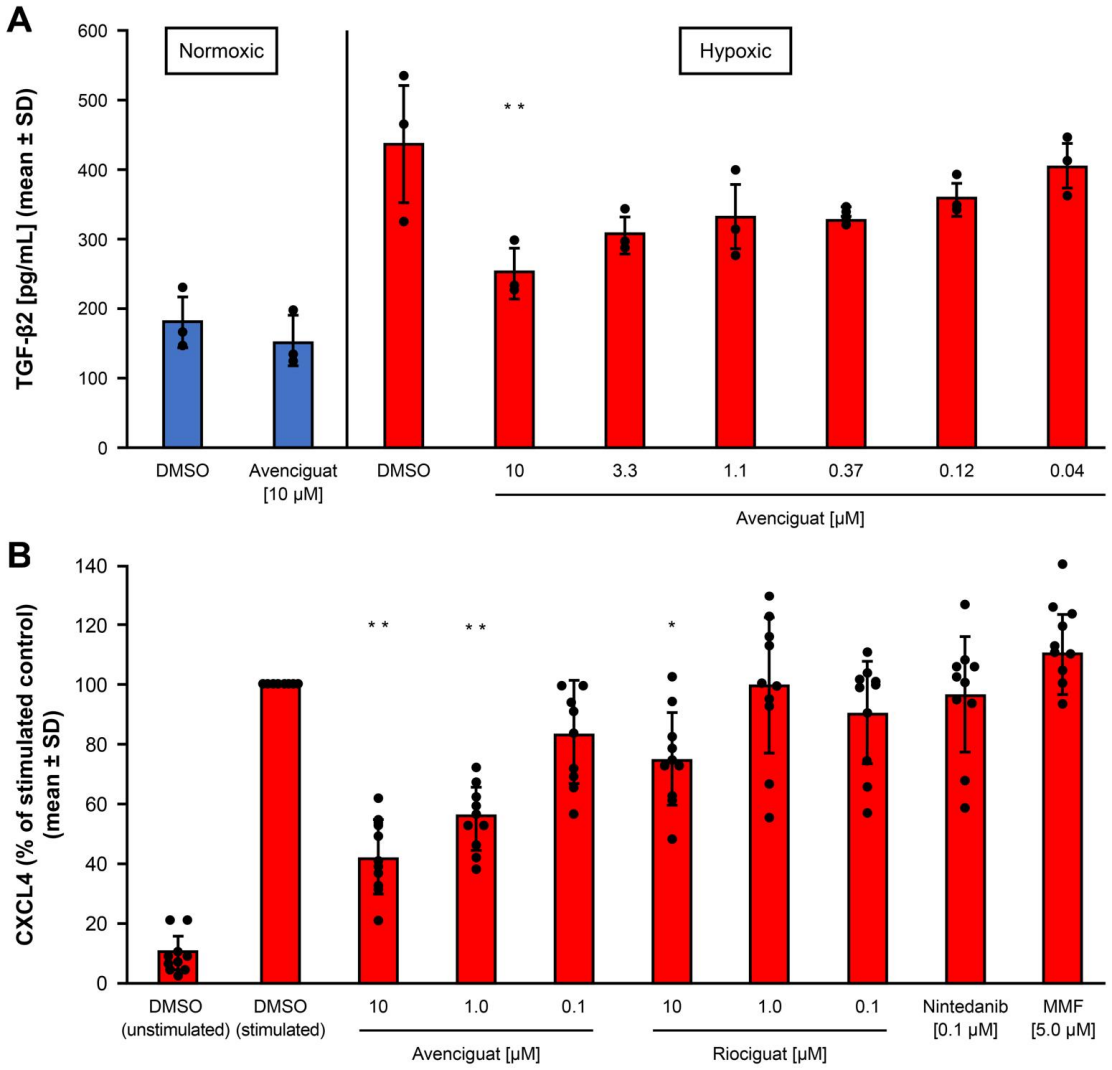
Effect of avenciguat on release of profibrotic and proinflammatory mediators in HMVEC-d and human PRP. (**A**) Assessing TGF-β2 levels: HMVEC-d were cultured in normoxic or hypoxic conditions with or without avenciguat and the levels of TGF-β2 were determined (mean ± s.d. of three separate experiments). *P *<* 0.05 *vs* DMSO control based on two-tailed Student’s *t-*test. (**B**) Assessing CXCL4 levels: human PRP was isolated and cultured in the presence or absence of avenciguat, riociguat, nintedanib or MMF, followed by activation with 10 mM ADP, and the level of CXCL4 was determined (mean ± s.d. of 10 separate experiments). **P *<* 0.01 and *P *<* 0.05 *vs* DMSO control based on two-tailed Student’s *t-*test. ADP: adenosine 5′-diphosphate; CXCL4: CXC chemokine family ligand 4; DMSO: dimethylsulfoxide; HMVEC-d: human microvascular endothelial cells-dermal; PRP: platelet-rich plasma

HMVEC-d produced higher levels of TGF-β2 when exposed to hypoxic *vs* normoxic conditions. After correcting for normoxic values, avenciguat inhibited hypoxia-induced TGF-β2 production in a dose-dependent manner, with 10 µM avenciguat resulting in a 61% reduction *vs* dimethylsulfoxide control (Fig. 1A).

### Avenciguat inhibits release of CXCL4 from activated human platelets

In activated PRP, which models the limited NO availability in SSc, avenciguat inhibited ADP-induced production of CXCL4 at concentrations of 10 and 1 µM (Fig. 1B). With riociguat, the effect was observed only at the highest dose (10 µM), whilst nintedanib and MMF were ineffective at the concentrations tested.

### Avenciguat ameliorates bleomycin-induced dermal and pulmonary fibrosis

**Figure 2.**
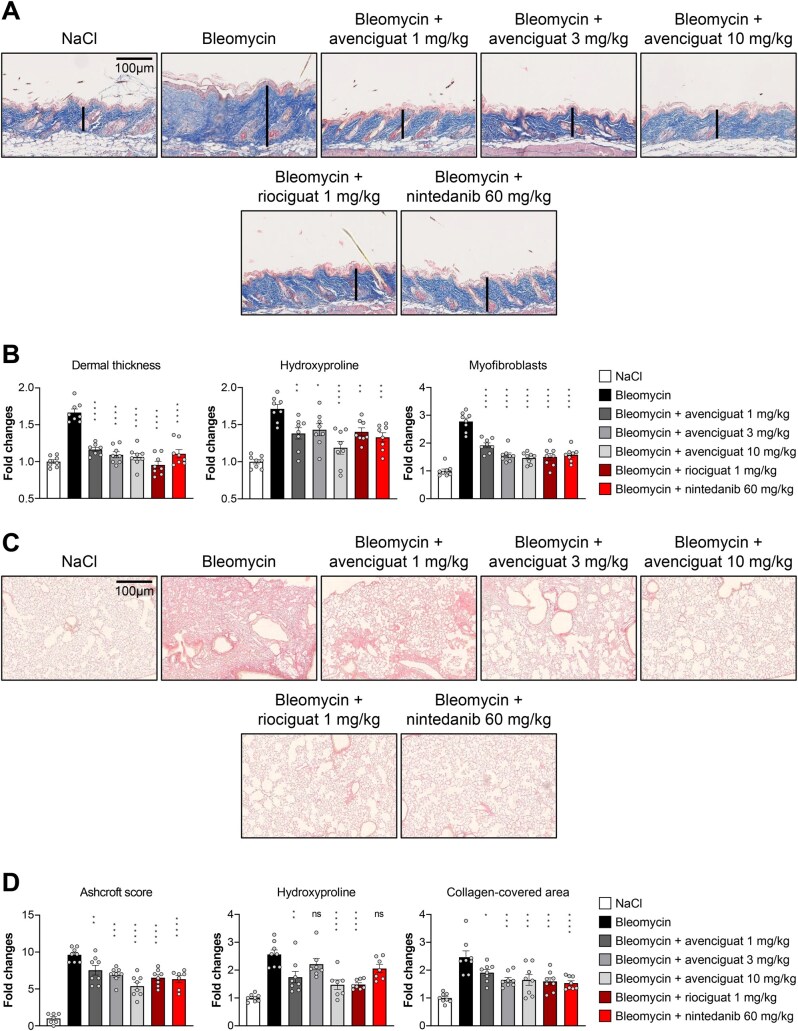
Treatment with avenciguat ameliorates bleomycin-induced dermal and pulmonary fibrosis. (A, B) Bleomycin-induced dermal fibrosis. (**A**) Trichrome staining at 100-fold magnification (*n* = 8 per group). (**B**) Dermal thickness, hydroxyproline content and myofibroblast count quantification. (C, D) Bleomycin-induced pulmonary fibrosis. (**C**) Sirius Red staining at 100-fold magnification (*n* = 8 per group). (**D**) Ashcroft score, hydroxyproline content and collagen-covered area quantification. Data are mean ± s.e.m. Statistical significance assessed by one-way analysis of variance with Dunnett’s multiple comparison. *P < 0.05, **P < 0.01, ***P < 0.001 and ****P < 0.0001 *vs* fibrotic control mice. NaCl: sodium chloride; ns: non-significant difference

Mice injected s.c. with bleomycin developed dermal fibrosis, whereas NaCl-treated control mice showed no fibrosis. Avenciguat treatment reduced dermal thickness, achieving levels comparable to those observed in NaCl-treated (control) mice (Fig. 2A and B). Avenciguat also reduced myofibroblast numbers and collagen deposition. No clear dose dependency was observed in the avenciguat groups, with similar effects seen at all doses. The effects observed were comparable to mice treated with nintedanib. Consistent with previous reports [7], riociguat also ameliorated bleomycin-induced dermal fibrosis. In this study, a number of genes related to collagen and fibronectin decreased following avenciguat treatment (Supplementary Table S1, available at *Rheumatology* online).

In bleomycin-treated animals, following avenciguat administration (Fig. 2C and D), dermal thickness, hydroxyproline and myofibroblast demonstrated a reduction of between 25 and 35%. The reduction in dermal thickness and myofibroblasts was seen to be dose dependent. Similar results were seen when assessing Ashcroft score, hydroxyproline and collagen-covered area.

### Avenciguat treatment modulates inflammatory and IFN-1 signalling

To further examine avenciguat’s effect in modulating fibrosis, RNA sequencing was performed on skin samples from mice with bleomycin-induced skin fibrosis. Using a cut-off of at least 2-fold (adjusted *P* *<* 0.05), 130 genes were up-regulated in the skin of bleomycin-treated mice (Supplementary Table S2, available at *Rheumatology* online). Of these, 12 were at least 1.5-fold (*P* *<* 0.01) down-modulated in avenciguat-treated animals (Supplementary Fig. S1A, available at *Rheumatology* online). Riociguat treatment down-regulated (≥1.5-fold; *P* *<* 0.01) four genes that were up-regulated in bleomycin-treated mice. These four genes were within the gene set identified in the avenciguat-treated group. The 12-gene set modified by avenciguat treatment is linked to IFN and inflammatory signalling (Supplementary Fig. S1A, available at *Rheumatology* online). Supplementary Fig. S1, available at *Rheumatology* online presents the top six processes associated with all genes down-modulated by avenciguat treatment (Supplementary Fig. S1B, available at *Rheumatology* online), the *z* scores normalized average expression of the 12-gene set (Supplementary Fig. S1C, available at *Rheumatology* online) and the up-regulation in patients with SSc *vs* healthy controls (Supplementary Fig. S1D, available at *Rheumatology* online), which was comparable when a generic IFN gene score was used (Supplementary Fig. S1E, available at *Rheumatology* online). Together, these underscore the clinical relevance of the avenciguat-modulated genes. Additional information regarding the results from these analyses and the genes that were down-regulated in bleomycin- and avenciguat-treated animals can be found in Supplementary Table S3, available at *Rheumatology* online.

## Discussion

In SSc, there is a high unmet need for disease-modifying agents, as therapies targeting the interplay of vascular disease, autoimmunity and fibrosis are not yet available. In preclinical models of SSc, the sGC–cGMP pathway has been shown to inhibit fibrosis [7]. Here, we report the first preclinical data demonstrating the efficacy of the sGC activator avenciguat in a murine model of SSc. In particular, these data demonstrate that sGC activators can dampen aberrant IFN signalling, a central pathway of autoimmunity and inflammation in SSc.

RNA sequencing revealed the impact of avenciguat on bleomycin-induced skin fibrosis in mice (Supplementary Fig. S1, available at *Rheumatology* online), showing down-regulated genes linked to IFN signalling, typically elevated in SSc patients, correlating with disease severity and treatment effectiveness [4]. Type I IFNs play a key role in the immune response by stimulating production of proteins that have antiviral, antiproliferative and immunomodulatory functions [4]. Thus, IFN signalling represents a potential therapeutic target for avenciguat. This rationale is supported by an ongoing clinical trial of the anti-IFN-α/β-receptor antibody anifrolumab in SSc (NCT05925803).

The data presented support the therapeutic potential of avenciguat in SSc through effects on multiple disease-relevant cell types, including microvascular endothelial cells and platelets in *in vitro* assays, and fibroblasts and IFN-1-releasing immune cells in bleomycin-induced fibrosis. Avenciguat reduced production of hypoxia-induced TGF-β2, a profibrotic cytokine, from microvascular endothelial cells. As hypoxia-driven production of reactive oxygen species is a key feature of SSc pathogenesis [2], the activity of avenciguat under hypoxic conditions is clinically relevant. Avenciguat also inhibited CXCL4 release from platelets. CXCL4 alters monocyte differentiation, driving proinflammatory and profibrotic phenotypes, and triggering a fibrotic cascade through extracellular matrix production and induction of myofibroblast differentiation [14]. Given the role of CXCL4 in bridging inflammation with fibrosis, inhibition of CXCL4 release may account for the effects on IFN signalling in the mouse model of bleomycin-induced fibrosis. However, further experiments are required to fully establish this relationship.

Avenciguat demonstrated specific effects not observed with the sGC stimulator, riociguat. Unlike riociguat, sGC activators are NO- and haem-independent, interacting directly with the haem binding site [8]. In environments of oxidative stress, such as fibrotic tissues of patients with SSc [2], a change in the redox of the haem molecule may render sGC non-responsive to sGC stimulators [8]. In bleomycin-induced fibrosis, avenciguat modulated genes associated with IFN-1 signalling. The inhibitory effect on the IFN-1 genes was significantly more pronounced than that observed with riociguat, suggesting that modulation of sGC with an sGC activator may lead to deeper inhibition of key SSc-associated pathways (i.e. IFN-1) than with an sGC stimulator. Avenciguat, unlike riociguat, also reduced CXCL4 release in activated PRP, a model that mimics limited NO availability. Together, these findings support the hypothesis that sGC activators may be more effective than sGC stimulators in environments of hypoxia and oxidative stress, as observed in the fibrotic tissues of patients with SSc.

Riociguat has been approved for the treatment of pulmonary arterial hypertension [9] and studied in patients with dcSSc [10]. In RISE-SSc, riociguat did not meet its primary endpoint of a 25% change from baseline in mRSS at week 52. However, potential efficacy signals were seen in secondary and exploratory endpoints, such as lung function improvements in patients with pre-existing interstitial lung disease and prevention of new RP and digital ulcer symptoms [10]. Given the potential advantages of sGC activators over sGC stimulators in conditions like SSc, and supported by the presented data, a Phase II trial investigating the safety and efficacy of avenciguat in patients with SSc is ongoing (NCT05559580). This trial is assessing the effect of avenciguat on fibrotic endpoints (lung fibrosis and skin thickening) and vascular injury endpoints (RP and digital ulcers). The trial employs unique inclusion criteria to enrich for patients with active SSc at higher risk of progression, inflammatory disease and significant vasculopathy. The primary endpoint is the rate of decline in forced vital capacity, and secondary endpoints include absolute change from baseline in mRSS at week 48.

## Supplementary Material

keaf109_Supplementary_Data

## Data Availability

Data sets for the analyses conducted in this manuscript are available from the corresponding author on reasonable request.
