# Supplementary material for: Avenciguat: a novel soluble guanylate cyclase activator that affects multiple cell types to inhibit IFN-1 signalling and fibrosis
Source: Rheumatology (Oxford). 2025 Feb 22;64(8):4738–43. doi: 10.1093/rheumatology/keaf109 (PMC12316371; doi:10.1093/rheumatology/keaf109)
Supplement: keaf109_Supplementary_Data [file keaf109_supplementary_data.docx]

**Supplement**

**Plain Language Summary**

Systemic sclerosis (SSc) is an autoimmune disease, a condition in which the body’s defence system, called the immune system, mistakenly damages normal tissue. In SSc, there is thickening or scarring of the skin and other tissues, known as fibrosis.

Soluble guanylate cyclase (sGC) is a naturally occurring substance that the body uses to produce another substance called cyclic guanosine monophosphate (cGMP), which can reduce fibrosis. sGC can be activated by stimulators and activators.

Drugs that stimulate sGC, such as riociguat, require a gas called nitric oxide (NO) to function optimally. In tissues with normal levels of oxygen, NO can bind to sGC and allows for efficient stimulation of sGC. However, in SSc, low levels of oxygen are available in tissues due to fibrosis. This makes sGC less responsive to NO. As such, sGC stimulators may not work as efficiently in low-oxygen environments. sGC activators, on the other hand, are a different type of drug that can function in low-oxygen environments.

Here, we present the results from studies in human tissues and animals showing the effects of the sGC activator avenciguat in SSc. Avenciguat was found to reduce the levels of molecules that are increased in low-oxygen environments. It also reduced the production of molecules that promote fibrosis. In tissue samples from mice that had skin and lung fibrosis, avenciguat reduced the levels of fibrosis.

These data suggest that avenciguat may target different aspects of SSc and is a potential future treatment for SSc.

**METHODS**

**RNA sequencing**

Total RNA was extracted from dermal tissue samples using the RNeasy Fibrous Tissue Mini Kit (QIAGEN, Hilden, Germany). A total of 80 ng RNA was used for library preparation using the NEBNext^®^ Ultra™ II Directional RNA Library Prep Kit for Illumina^®^ (New England Laboratories, Ipswich, MA, USA) and subsequent RNA sequencing. RNA sequencing was performed on the Illumina NovaSeq 6000 platform (Illumina, San Diego, CA, USA) with a 107 base-pair cycle single-end read.

**Gene expression analysis**

The enrichR enrichment analysis tool was applied for gene set enrichment analysis using the Reactome 2022 gene set library as a reference. Gene sets with an adjusted *P* < 0.05 were considered statistically significant. A gene score was calculated using the rank-based single-sample scoring method (singscore) approach (bioconductor.org) of the genes modulated by avenciguat and leveraged to published human gene expression data obtained from healthy control and SSc skin biopsies [1].

Sequenced reads were mapped against reference genome GRCm38.p4 using the STAR mapping tool version 2.5.2a. Raw read counts were calculated using Subread. Fold changes and their respective significance were computed based on the read counts obtained for each gene using R and Bioconductor packages edgeR, DESeq2 or voom in conjunction with limma. Adjustment of *P*-values for multiple testing was performed according to Benjamini and Hochberg.

**RESULTS**

In total, 44 genes were at least 1.5-fold decreased in avenciguat-treated animals, including 12 genes that were at least 2-fold up-regulated in bleomycin-treated animals compared with controls (Supplementary Fig. S1A). Analysis of the 44 down-regulated genes using the Reactome database demonstrated that the top six pathways with significant enrichment corresponded to immune/inflammatory and IFN-1 signalling pathways (Supplementary Fig. S1B). Four of the 12 genes increased by bleomycin treatment and down-regulated by avenciguat were also down-regulated by riociguat. To assess differences in gene expression between the avenciguat and riociguat groups, a comparison of the z-score normalized average expression of the 12-gene set was performed. Avenciguat treatment resulted in an overall greater decrease in gene expression *vs* riociguat (Supplementary Fig. S1C). This result suggests that at a molecular level, avenciguat leads to deeper modulation of immune/inflammatory and IFN-1 pathways *in vivo* *vs* riociguat.

Finally, a gene score was calculated for the avenciguat-modulated gene set and analysed against the gene expression data set of SSc and healthy control skin biopsies [2]. Supplementary Fig. S1D shows significant up-regulation of the 12-gene set in SSc patients *vs* healthy controls. Using a generic IFN composite score (which comprises IFIT1, IFIT2, IFIT3, IFI44, IFI44L, IFI6, ISG15, LY6E, MX1, MX2, OAS1, OAS2, OAS3, RSAD2, SIGLEC1, XAF1, EPSTI1 and HERC5) resulted in an increased IFN gene score in SSc patients compared with healthy controls (Supplementary Fig. S1E).

**Supplementary Table S1. Expression of genes encoding collagen, fibronectin and COMP**

| Gene symbol | Bleomycin.vs.  NaCl_logFC | Bleomycin.vs.  NaCl_P.Value | Bleomycin.vs.  NaCl_adj.P.Value | Avenciguat_  Bleomycin.vs.  Bleomycin_logFC | Avenciguat_  Bleomycin.vs.  Bleomycin_  P.Value | Avenciguat_  Bleomycin.vs.  Bleomycin_adj.P.Value | Riociguat_  Bleomycin.vs.  Bleomycin_logFC | Riociguat_  Bleomycin.vs.  Bleomycin_  P.Value | Riociguat_  Bleomycin.vs.  Bleomycin_adj.P.Value |
| --- | --- | --- | --- | --- | --- | --- | --- | --- | --- |
| Col10a1 | 0.52 | 0.09 | 0.27 | -0.26 | 0.40 | 0.97 | -0.30 | 0.32 | 1.00 |
| Col11a1 | -0.96 | 0.03 | 0.18 | -0.05 | 0.92 | 1.00 | -0.52 | 0.25 | 1.00 |
| Col11a2 | -1.00 | 0.14 | 0.35 | 0.15 | 0.82 | 1.00 | -0.50 | 0.46 | 1.00 |
| Col12a1 | 0.77 | 0.05 | 0.21 | -0.40 | 0.29 | 0.89 | -0.33 | 0.38 | 1.00 |
| Col13a1 | -0.86 | 0.12 | 0.32 | 0.08 | 0.89 | 1.00 | -0.63 | 0.26 | 1.00 |
| Col14a1 | -0.13 | 0.62 | 0.70 | -0.21 | 0.44 | 1.00 | 0.38 | 0.16 | 1.00 |
| Col15a1 | 0.82 | 0.01 | 0.14 | -0.60 | 0.07 | 0.85 | 0.02 | 0.95 | 1.00 |
| Col16a1 | 0.14 | 0.55 | 0.65 | -0.37 | 0.13 | 0.85 | -0.12 | 0.62 | 1.00 |
| Col17a1 | 1.45 | 0.02 | 0.15 | -0.69 | 0.25 | 0.86 | 0.33 | 0.58 | 1.00 |
| Col18a1 | 0.59 | 0.12 | 0.33 | -0.36 | 0.34 | 0.93 | -0.16 | 0.67 | 1.00 |
| Col19a1 | -0.45 | 0.45 | 0.57 | 0.09 | 0.88 | 1.00 | -0.45 | 0.45 | 1.00 |
| Col1a1 | 1.46 | 0.04 | 0.19 | -1.59 | 0.02 | 0.85 | -1.03 | 0.14 | 1.00 |
| Col1a2 | 1.59 | 0.01 | 0.14 | -1.33 | 0.04 | 0.85 | -0.53 | 0.40 | 1.00 |
| Col20a1 | -0.07 | 0.71 | 0.77 | 0.14 | 0.48 | 1.00 | 0.20 | 0.30 | 1.00 |
| Col22a1 | -0.66 | 0.29 | 0.47 | 0.25 | 0.69 | 1.00 | -0.22 | 0.72 | 1.00 |
| Col23a1 | 0.55 | 0.01 | 0.14 | -0.27 | 0.22 | 0.85 | 0.13 | 0.55 | 1.00 |
| Col24a1 | -0.61 | 0.17 | 0.38 | 0.33 | 0.46 | 1.00 | -0.08 | 0.85 | 1.00 |
| Col25a1 | -0.54 | 0.46 | 0.58 | 0.30 | 0.68 | 1.00 | -0.84 | 0.25 | 1.00 |
| Col26a1 | -1.21 | 0.09 | 0.28 | 0.34 | 0.63 | 1.00 | -0.40 | 0.57 | 1.00 |
| Col27a1 | -0.43 | 0.05 | 0.22 | 0.18 | 0.41 | 0.98 | -0.13 | 0.56 | 1.00 |
| Col28a1 | -0.29 | 0.33 | 0.50 | 0.38 | 0.20 | 0.85 | 0.35 | 0.23 | 1.00 |
| Col2a1 | -1.11 | 0.25 | 0.45 | 0.33 | 0.73 | 1.00 | -0.75 | 0.44 | 1.00 |
| Col3a1 | 1.61 | 0.01 | 0.14 | -1.23 | 0.06 | 0.85 | -0.23 | 0.72 | 1.00 |
| Col4a1 | 1.16 | 0.01 | 0.14 | -0.76 | 0.10 | 0.85 | -0.11 | 0.81 | 1.00 |
| Col4a2 | 0.97 | 0.03 | 0.17 | -0.53 | 0.23 | 0.86 | 0.06 | 0.89 | 1.00 |
| Col4a3 | -0.72 | 0.05 | 0.22 | 0.26 | 0.47 | 1.00 | -0.01 | 0.98 | 1.00 |
| Col4a4 | -0.50 | 0.00 | 0.10 | 0.19 | 0.27 | 0.87 | 0.07 | 0.66 | 1.00 |
| Col4a5 | 0.11 | 0.48 | 0.59 | 0.01 | 0.96 | 1.00 | 0.02 | 0.91 | 1.00 |
| Col4a6 | 0.04 | 0.78 | 0.82 | 0.14 | 0.40 | 0.97 | 0.26 | 0.11 | 1.00 |
| Col5a1 | 0.84 | 0.05 | 0.21 | -0.71 | 0.10 | 0.85 | -0.30 | 0.47 | 1.00 |
| Col5a2 | 1.18 | 0.01 | 0.13 | -0.71 | 0.12 | 0.85 | -0.17 | 0.72 | 1.00 |
| Col5a3 | 0.72 | 0.05 | 0.21 | -0.37 | 0.31 | 0.90 | -0.32 | 0.37 | 1.00 |
| Col6a1 | 0.99 | 0.04 | 0.20 | -0.64 | 0.18 | 0.85 | 0.04 | 0.93 | 1.00 |
| Col6a2 | 0.92 | 0.06 | 0.22 | -0.68 | 0.15 | 0.85 | -0.18 | 0.70 | 1.00 |
| Col6a3 | 0.48 | 0.31 | 0.48 | -0.47 | 0.32 | 0.91 | 0.09 | 0.85 | 1.00 |
| Col6a4 | -1.04 | 0.29 | 0.47 | 0.21 | 0.83 | 1.00 | -0.57 | 0.56 | 1.00 |
| Col6a5 | -0.28 | 0.44 | 0.57 | 0.49 | 0.19 | 0.85 | 0.03 | 0.94 | 1.00 |
| Col6a6 | -0.69 | 0.01 | 0.14 | 0.31 | 0.26 | 0.87 | 0.05 | 0.85 | 1.00 |
| Col7a1 | 0.40 | 0.12 | 0.31 | -0.10 | 0.68 | 1.00 | -0.06 | 0.82 | 1.00 |
| Col8a1 | 1.60 | 0.00 | 0.01 | -0.08 | 0.83 | 1.00 | 0.04 | 0.92 | 1.00 |
| Col8a2 | 0.28 | 0.05 | 0.21 | -0.27 | 0.06 | 0.85 | -0.36 | 0.01 | 1.00 |
| Col9a1 | -0.41 | 0.72 | 0.78 | -0.09 | 0.94 | 1.00 | -1.26 | 0.28 | 1.00 |
| Col9a2 | -0.72 | 0.46 | 0.58 | 0.45 | 0.65 | 1.00 | -0.30 | 0.76 | 1.00 |
| Col9a3 | -0.73 | 0.16 | 0.37 | 0.26 | 0.62 | 1.00 | -0.33 | 0.53 | 1.00 |
| Comp | -0.77 | 0.19 | 0.40 | 0.21 | 0.72 | 1.00 | -0.12 | 0.83 | 1.00 |
| Fn1 | 1.50 | 0.02 | 0.14 | -0.60 | 0.33 | 0.92 | 0.71 | 0.24 | 1.00 |

**Supplementary Table S2. List of 130 genes up-regulated in the bleomycin-induced skin fibrosis model and their changes upon treatment with riociguat and avenciguat**

| Gene_symbol | Bleomycin.vs.NaCl_logFC | Bleomycin.vs.NaCl_P.Value | Bleomycin.vs.NaCl_adj.P.Value | Avenciguat_Bleomycin.vs.Bleomycin_logFC | Avenciguat_Bleomycin.vs.Bleomycin_P.Value | Avenciguat_Bleomycin.vs.Bleomycin_adj.P.Value | Riociguat_Bleomycin.vs.Bleomycin_logFC | Riociguat_Bleomycin.vs.Bleomycin_P.Value | Riociguat_Bleomycin.vs.Bleomycin_adj.P.Value |
| --- | --- | --- | --- | --- | --- | --- | --- | --- | --- |
| 5430431A17Rik | 2.08 | 3.79E-05 | 0.01 | -0.52 | 0.27 | 0.88 | -0.09 | 0.85 | 1.00 |
| Abra | 1.62 | 4.79E-05 | 0.01 | -0.30 | 0.42 | 0.99 | 0.29 | 0.44 | 1.00 |
| Actc1 | 2.56 | 4.71E-05 | 0.01 | -0.62 | 0.29 | 0.89 | -0.43 | 0.47 | 1.00 |
| Ankrd1 | 2.63 | 4.57E-07 | 0.00 | -0.40 | 0.40 | 0.98 | -0.72 | 0.13 | 1.00 |
| Aplnr | 1.06 | 4.42E-05 | 0.01 | -0.52 | 0.04 | 0.85 | -0.30 | 0.21 | 1.00 |
| B4galt5 | 1.21 | 2.06E-05 | 0.00 | -0.28 | 0.29 | 0.89 | -0.12 | 0.66 | 1.00 |
| Bst2 | 1.56 | 2.82E-07 | 0.00 | -0.51 | 0.07 | 0.85 | -0.18 | 0.52 | 1.00 |
| C2 | 1.33 | 1.60E-06 | 0.00 | -0.32 | 0.22 | 0.85 | 0.15 | 0.55 | 1.00 |
| C3ar1 | 1.83 | 5.28E-06 | 0.00 | -0.56 | 0.14 | 0.85 | -0.11 | 0.78 | 1.00 |
| Casq2 | 1.66 | 6.68E-06 | 0.00 | -0.06 | 0.87 | 1.00 | -0.41 | 0.23 | 1.00 |
| Ccl2 | 2.09 | 1.30E-06 | 0.00 | -0.44 | 0.27 | 0.87 | -0.05 | 0.91 | 1.00 |
| Ccl7 | 2.67 | 2.89E-08 | 3.68E-05 | -0.76 | 0.08 | 0.85 | -0.38 | 0.38 | 1.00 |
| Ccr5 | 2.23 | 4.93E-08 | 5.23E-05 | -0.54 | 0.14 | 0.85 | -0.38 | 0.30 | 1.00 |
| Cd180 | 1.03 | 4.19E-05 | 0.01 | -0.35 | 0.14 | 0.85 | -0.02 | 0.92 | 1.00 |
| Cd274 | 1.20 | 4.68E-06 | 0.00 | -0.67 | 0.01 | 0.85 | -0.58 | 0.02 | 1.00 |
| Cd53 | 1.98 | 2.19E-05 | 0.01 | -1.00 | 0.02 | 0.85 | -0.26 | 0.54 | 1.00 |
| Cd93 | 1.54 | 4.72E-05 | 0.01 | -0.45 | 0.20 | 0.85 | 0.05 | 0.88 | 1.00 |
| Chrna1 | 1.86 | 1.14E-06 | 0.00 | 0.13 | 0.70 | 1.00 | -0.02 | 0.95 | 1.00 |
| Chrnb1 | 1.21 | 1.69E-05 | 0.00 | -0.08 | 0.75 | 1.00 | 0.00 | 1.00 | 1.00 |
| Clec4a1 | 1.45 | 4.81E-05 | 0.01 | -0.57 | 0.09 | 0.85 | -0.14 | 0.68 | 1.00 |
| Cmpk2 | 1.21 | 9.11E-06 | 0.00 | -0.77 | 0.00 | 0.85 | -0.61 | 0.02 | 1.00 |
| Col8a1 | 1.60 | 2.31E-05 | 0.01 | -0.08 | 0.83 | 1.00 | 0.04 | 0.92 | 1.00 |
| Ctss | 2.59 | 1.03E-05 | 0.00 | -0.81 | 0.14 | 0.85 | -0.23 | 0.68 | 1.00 |
| Cxcl10 | 1.46 | 4.41E-07 | 0.00 | -0.76 | 0.00 | 0.85 | -0.72 | 0.01 | 1.00 |
| Cybb | 1.55 | 4.84E-06 | 0.00 | -0.55 | 0.08 | 0.85 | -0.23 | 0.46 | 1.00 |
| Ddx58 | 1.48 | 1.92E-06 | 0.00 | -0.54 | 0.06 | 0.85 | -0.24 | 0.39 | 1.00 |
| Ddx60 | 1.50 | 1.02E-07 | 9.31E-05 | -0.55 | 0.03 | 0.85 | -0.26 | 0.30 | 1.00 |
| Dtna | 1.35 | 2.85E-05 | 0.01 | -0.09 | 0.77 | 1.00 | 0.17 | 0.57 | 1.00 |
| Dtx3l | 2.08 | 2.14E-06 | 0.00 | -0.82 | 0.04 | 0.85 | -0.69 | 0.09 | 1.00 |
| Eda2r | 2.09 | 4.11E-09 | 9.39E-06 | -0.34 | 0.28 | 0.88 | -0.20 | 0.52 | 1.00 |
| Eif2ak2 | 1.33 | 3.94E-08 | 4.45E-05 | -0.45 | 0.04 | 0.85 | -0.38 | 0.08 | 1.00 |
| Epsti1 | 1.09 | 1.93E-05 | 0.00 | -0.51 | 0.04 | 0.85 | -0.25 | 0.29 | 1.00 |
| F830016B08Rik | 2.40 | 1.39E-06 | 0.00 | -0.69 | 0.13 | 0.85 | -0.11 | 0.81 | 1.00 |
| Gbp2 | 1.76 | 5.41E-06 | 0.00 | -0.75 | 0.04 | 0.85 | -0.24 | 0.50 | 1.00 |
| Gbp3 | 1.48 | 2.71E-08 | 3.68E-05 | -0.36 | 0.13 | 0.85 | -0.03 | 0.90 | 1.00 |
| Gbp4 | 1.17 | 9.40E-07 | 0.00 | -0.30 | 0.16 | 0.85 | -0.23 | 0.29 | 1.00 |
| Gbp6 | 1.81 | 1.22E-08 | 1.90E-05 | -0.74 | 0.01 | 0.85 | -0.55 | 0.05 | 1.00 |
| Gbp7 | 1.48 | 2.88E-06 | 0.00 | -0.53 | 0.07 | 0.85 | -0.03 | 0.92 | 1.00 |
| Gbp9 | 1.34 | 3.96E-06 | 0.00 | -0.43 | 0.11 | 0.85 | -0.02 | 0.93 | 1.00 |
| Gm4841 | 2.27 | 1.80E-06 | 0.00 | -0.54 | 0.22 | 0.85 | -0.74 | 0.09 | 1.00 |
| Gm4951 | 1.91 | 4.25E-09 | 9.39E-06 | -0.74 | 0.01 | 0.85 | -0.64 | 0.03 | 1.00 |
| Gm5431 | 1.02 | 4.05E-05 | 0.01 | -0.38 | 0.10 | 0.85 | -0.07 | 0.76 | 1.00 |
| Gm8995 | 1.44 | 2.97E-05 | 0.01 | -0.51 | 0.11 | 0.85 | 0.08 | 0.81 | 1.00 |
| Gvin-ps2 | 1.38 | 5.32E-07 | 0.00 | -0.49 | 0.05 | 0.85 | 0.05 | 0.84 | 1.00 |
| Gvin-ps4 | 1.27 | 3.30E-06 | 0.00 | -0.46 | 0.07 | 0.85 | 0.06 | 0.80 | 1.00 |
| Gvin1 | 1.81 | 9.23E-07 | 0.00 | -0.58 | 0.09 | 0.85 | 0.07 | 0.83 | 1.00 |
| Gvin2 | 1.83 | 8.46E-07 | 0.00 | -0.59 | 0.08 | 0.85 | 0.06 | 0.85 | 1.00 |
| Herc6 | 1.04 | 4.16E-06 | 0.00 | -0.50 | 0.02 | 0.85 | -0.34 | 0.11 | 1.00 |
| Hspb7 | 2.68 | 8.26E-06 | 0.00 | -0.14 | 0.81 | 1.00 | 0.02 | 0.97 | 1.00 |
| Ifi203 | 1.46 | 1.18E-06 | 0.00 | -0.64 | 0.02 | 0.85 | -0.14 | 0.61 | 1.00 |
| Ifi204 | 1.58 | 1.13E-06 | 0.00 | -0.78 | 0.01 | 0.85 | -0.20 | 0.51 | 1.00 |
| Ifi207 | 1.24 | 2.24E-05 | 0.01 | -0.53 | 0.06 | 0.85 | 0.17 | 0.53 | 1.00 |
| Ifi213 | 1.70 | 2.31E-07 | 0.00 | -0.71 | 0.02 | 0.85 | -0.45 | 0.13 | 1.00 |
| Ifi27l2a | 2.42 | 3.66E-05 | 0.01 | -1.01 | 0.07 | 0.85 | 0.26 | 0.64 | 1.00 |
| Ifi44 | 1.99 | 4.37E-10 | 1.29E-06 | -0.77 | 0.01 | 0.85 | -0.86 | 0.00 | 1.00 |
| Ifi47 | 1.75 | 8.10E-07 | 0.00 | -0.73 | 0.03 | 0.85 | -0.36 | 0.27 | 1.00 |
| Ifih1 | 1.38 | 3.11E-06 | 0.00 | -0.66 | 0.02 | 0.85 | -0.15 | 0.58 | 1.00 |
| Ifit1 | 3.00 | 2.23E-12 | 5.92E-08 | -1.09 | 0.00 | 0.85 | -0.63 | 0.08 | 1.00 |
| Ifit2 | 1.46 | 1.76E-06 | 0.00 | -0.64 | 0.02 | 0.85 | -0.17 | 0.53 | 1.00 |
| Ifit3 | 2.58 | 1.53E-11 | 1.35E-07 | -0.87 | 0.01 | 0.85 | -0.60 | 0.06 | 1.00 |
| Ifit3b | 2.35 | 3.41E-10 | 1.20E-06 | -0.82 | 0.01 | 0.85 | -0.62 | 0.06 | 1.00 |
| Igf2 | 2.24 | 1.07E-05 | 0.00 | -0.48 | 0.31 | 0.91 | -0.37 | 0.44 | 1.00 |
| Igtp | 1.70 | 6.40E-06 | 0.00 | -0.63 | 0.07 | 0.85 | -0.36 | 0.30 | 1.00 |
| Iigp1 | 2.42 | 6.57E-08 | 6.21E-05 | -0.99 | 0.01 | 0.85 | -0.82 | 0.04 | 1.00 |
| Irf7 | 1.70 | 1.86E-06 | 0.00 | -0.72 | 0.03 | 0.85 | -0.17 | 0.61 | 1.00 |
| Irf9 | 1.48 | 3.52E-05 | 0.01 | -0.67 | 0.05 | 0.85 | -0.41 | 0.22 | 1.00 |
| Irgm1 | 1.59 | 1.74E-07 | 0.00 | -0.64 | 0.02 | 0.85 | -0.27 | 0.32 | 1.00 |
| Irgm2 | 2.17 | 1.25E-06 | 0.00 | -0.70 | 0.09 | 0.85 | -0.44 | 0.28 | 1.00 |
| Isg15 | 2.16 | 4.01E-08 | 4.45E-05 | -0.81 | 0.02 | 0.85 | -0.71 | 0.04 | 1.00 |
| Itgam | 1.46 | 4.33E-05 | 0.01 | -0.37 | 0.26 | 0.87 | 0.02 | 0.94 | 1.00 |
| Lgals3bp | 2.16 | 1.56E-06 | 0.00 | -0.91 | 0.03 | 0.85 | -0.06 | 0.89 | 1.00 |
| Lgals9 | 1.88 | 3.86E-06 | 0.00 | -0.85 | 0.02 | 0.85 | -0.29 | 0.43 | 1.00 |
| Lilrb4a | 1.84 | 1.68E-05 | 0.00 | -0.56 | 0.16 | 0.85 | 0.08 | 0.83 | 1.00 |
| Lrrc2 | 1.32 | 3.32E-05 | 0.01 | 0.20 | 0.50 | 1.00 | 0.17 | 0.56 | 1.00 |
| Ly6c2 | 1.50 | 4.42E-06 | 0.00 | -0.61 | 0.05 | 0.85 | 0.03 | 0.92 | 1.00 |
| Mndal | 1.37 | 4.83E-06 | 0.00 | -0.43 | 0.12 | 0.85 | 0.12 | 0.67 | 1.00 |
| Ms4a4c | 3.12 | 3.61E-10 | 1.20E-06 | -1.00 | 0.02 | 0.85 | -0.53 | 0.21 | 1.00 |
| Ms4a6b | 1.17 | 3.39E-05 | 0.01 | -0.41 | 0.12 | 0.85 | -0.09 | 0.73 | 1.00 |
| Ms4a7 | 1.38 | 8.27E-06 | 0.00 | -0.39 | 0.18 | 0.85 | -0.19 | 0.51 | 1.00 |
| Mx1 | 2.40 | 8.31E-11 | 5.50E-07 | -1.10 | 0.00 | 0.85 | -0.98 | 0.00 | 1.00 |
| Myf6 | 2.08 | 3.18E-06 | 0.00 | -0.72 | 0.08 | 0.85 | -0.26 | 0.52 | 1.00 |
| Mymk | 2.39 | 3.03E-05 | 0.01 | -0.69 | 0.20 | 0.85 | -0.83 | 0.13 | 1.00 |
| Myog | 2.11 | 9.28E-07 | 0.00 | -0.46 | 0.24 | 0.86 | -0.46 | 0.24 | 1.00 |
| Nr1d1 | 2.35 | 3.11E-05 | 0.01 | -0.59 | 0.26 | 0.87 | 1.25 | 0.02 | 1.00 |
| Oas1a | 1.74 | 8.81E-09 | 1.46E-05 | -0.55 | 0.04 | 0.85 | -0.37 | 0.17 | 1.00 |
| Oas1b | 1.15 | 3.42E-06 | 0.00 | -0.56 | 0.02 | 0.85 | -0.41 | 0.08 | 1.00 |
| Oas1g | 1.41 | 2.40E-06 | 0.00 | -0.49 | 0.07 | 0.85 | -0.30 | 0.27 | 1.00 |
| Oas2 | 1.68 | 4.03E-08 | 4.45E-05 | -0.60 | 0.03 | 0.85 | -0.19 | 0.49 | 1.00 |
| Oas3 | 1.71 | 7.22E-07 | 0.00 | -0.56 | 0.08 | 0.85 | -0.46 | 0.15 | 1.00 |
| Oasl2 | 2.66 | 2.12E-10 | 1.11E-06 | -0.92 | 0.01 | 0.85 | -0.50 | 0.16 | 1.00 |
| Parp11 | 1.12 | 6.95E-06 | 0.00 | -0.50 | 0.03 | 0.85 | -0.57 | 0.02 | 1.00 |
| Parp12 | 1.54 | 7.60E-06 | 0.00 | -0.69 | 0.03 | 0.85 | -0.25 | 0.43 | 1.00 |
| Parp14 | 1.76 | 7.95E-06 | 0.00 | -0.64 | 0.08 | 0.85 | -0.21 | 0.57 | 1.00 |
| Pctp | 1.20 | 3.54E-06 | 0.00 | -0.26 | 0.28 | 0.88 | -0.13 | 0.57 | 1.00 |
| Phf11d | 1.30 | 1.04E-06 | 0.00 | -0.45 | 0.06 | 0.85 | -0.16 | 0.50 | 1.00 |
| Pik3ap1 | 1.22 | 9.34E-06 | 0.00 | -0.40 | 0.12 | 0.85 | -0.22 | 0.39 | 1.00 |
| Plek | 1.47 | 7.97E-07 | 0.00 | -0.41 | 0.13 | 0.85 | -0.09 | 0.74 | 1.00 |
| Pon1 | 1.76 | 6.34E-06 | 0.00 | -0.52 | 0.15 | 0.85 | -0.34 | 0.35 | 1.00 |
| Rbm24 | 1.64 | 4.19E-05 | 0.01 | -0.18 | 0.63 | 1.00 | 0.21 | 0.58 | 1.00 |
| Rnf213 | 2.14 | 2.26E-05 | 0.01 | -0.85 | 0.07 | 0.85 | -0.16 | 0.73 | 1.00 |
| Rsad2 | 2.64 | 8.07E-12 | 1.07E-07 | -1.17 | 0.00 | 0.85 | -1.13 | 0.00 | 0.97 |
| Rtp4 | 2.25 | 2.51E-10 | 1.11E-06 | -0.66 | 0.03 | 0.85 | -0.32 | 0.29 | 1.00 |
| Samd9l | 1.32 | 2.39E-05 | 0.01 | -0.49 | 0.09 | 0.85 | 0.09 | 0.76 | 1.00 |
| Sdc3 | 1.83 | 4.28E-05 | 0.01 | -0.68 | 0.11 | 0.85 | -0.15 | 0.72 | 1.00 |
| Serpinb1a | 1.56 | 2.33E-05 | 0.01 | -0.05 | 0.89 | 1.00 | 0.43 | 0.22 | 1.00 |
| Slfn1 | 2.45 | 7.76E-09 | 1.37E-05 | -0.69 | 0.07 | 0.85 | -0.44 | 0.24 | 1.00 |
| Slfn2 | 2.09 | 6.33E-07 | 0.00 | -0.66 | 0.08 | 0.85 | -0.25 | 0.50 | 1.00 |
| Slfn4 | 2.90 | 2.31E-09 | 6.11E-06 | -0.83 | 0.05 | 0.85 | -0.22 | 0.60 | 1.00 |
| Slfn8 | 1.78 | 2.91E-08 | 3.68E-05 | -0.61 | 0.03 | 0.85 | -0.14 | 0.63 | 1.00 |
| Sln | 4.28 | 7.31E-09 | 1.37E-05 | -0.28 | 0.67 | 1.00 | 0.38 | 0.56 | 1.00 |
| Sp100 | 1.25 | 6.79E-07 | 0.00 | -0.37 | 0.11 | 0.85 | -0.04 | 0.87 | 1.00 |
| Stat1 | 1.34 | 4.84E-06 | 0.00 | -0.62 | 0.02 | 0.85 | -0.43 | 0.12 | 1.00 |
| Stat2 | 1.35 | 4.61E-06 | 0.00 | -0.66 | 0.02 | 0.85 | -0.33 | 0.23 | 1.00 |
| Tgtp1 | 1.18 | 2.46E-06 | 0.00 | -0.39 | 0.09 | 0.85 | -0.40 | 0.08 | 1.00 |
| Tgtp2 | 1.38 | 1.10E-06 | 0.00 | -0.43 | 0.10 | 0.85 | -0.39 | 0.14 | 1.00 |
| Themis2 | 1.43 | 6.31E-06 | 0.00 | -0.55 | 0.06 | 0.85 | -0.11 | 0.71 | 1.00 |
| Tlr2 | 1.11 | 5.08E-05 | 0.01 | -0.29 | 0.26 | 0.87 | -0.28 | 0.27 | 1.00 |
| Tlr3 | 1.21 | 1.23E-05 | 0.00 | -0.44 | 0.09 | 0.85 | -0.19 | 0.46 | 1.00 |
| Tlr9 | 1.18 | 4.80E-05 | 0.01 | -0.53 | 0.05 | 0.85 | -0.15 | 0.58 | 1.00 |
| Trim25 | 1.67 | 3.14E-05 | 0.01 | -0.62 | 0.10 | 0.85 | -0.28 | 0.45 | 1.00 |
| Trim30a | 2.19 | 5.36E-08 | 5.46E-05 | -0.90 | 0.01 | 0.85 | -0.36 | 0.31 | 1.00 |
| Trim30d | 1.56 | 5.62E-08 | 5.52E-05 | -0.73 | 0.01 | 0.85 | -0.32 | 0.22 | 1.00 |
| Trim34a | 1.30 | 2.20E-08 | 3.24E-05 | -0.46 | 0.03 | 0.85 | -0.24 | 0.25 | 1.00 |
| Trim72 | 1.58 | 1.34E-05 | 0.00 | -0.15 | 0.65 | 1.00 | -0.04 | 0.91 | 1.00 |
| Uba7 | 1.00 | 4.47E-05 | 0.01 | -0.32 | 0.16 | 0.85 | -0.03 | 0.91 | 1.00 |
| Ube2l6 | 1.91 | 4.48E-06 | 0.00 | -0.64 | 0.10 | 0.85 | -0.72 | 0.06 | 1.00 |
| Usp18 | 1.83 | 6.61E-09 | 1.35E-05 | -0.79 | 0.01 | 0.85 | -0.67 | 0.02 | 1.00 |
| Xaf1 | 1.63 | 2.29E-07 | 0.00 | -0.51 | 0.08 | 0.85 | -0.20 | 0.48 | 1.00 |
| Zbp1 | 1.71 | 1.24E-07 | 0.00 | -0.44 | 0.13 | 0.85 | 0.03 | 0.92 | 1.00 |
| Zdbf2 | 1.24 | 2.57E-05 | 0.01 | -0.11 | 0.69 | 1.00 | -0.32 | 0.24 | 1.00 |

**Supplementary Table S3. List of 44 down-regulated genes in bleomycin- and avenciguat-treated mice**

| Gene_symbol | Bleomycin.vs.NaCl_logFC | Bleomycin.vs.NaCl_P.Value | Bleomycin.vs.NaCl_adj.P.Value | Avenciguat_Bleomycin.vs.Bleomycin_logFC | Avenciguat_Bleomycin.vs.Bleomycin_P.Value | Avenciguat_Bleomycin.vs.Bleomycin_adj.P.Value | Riociguat_Bleomycin.vs.Bleomycin_logFC | Riociguat_Bleomycin.vs.Bleomycin_P.Value | Riociguat_Bleomycin.vs.Bleomycin_adj.P.Value |
| --- | --- | --- | --- | --- | --- | --- | --- | --- | --- |
| Scand1 | 0.02 | 0.97 | 0.98 | -1.95 | 0.00 | 0.85 | -2.49 | 8.88E-05 | 0.47 |
| Gm25848 | 0.48 | 0.35 | 0.51 | -1.37 | 0.01 | 0.85 | -1.19 | 0.02 | 1.00 |
| 1110065P20Rik | -0.10 | 0.78 | 0.82 | -1.31 | 0.00 | 0.85 | -0.56 | 0.12 | 1.00 |
| Rsad2 | 2.64 | 8.07E-12 | 1.07E-07 | -1.17 | 0.00 | 0.85 | -1.13 | 0.00 | 0.97 |
| Mx1 | 2.40 | 8.31E-11 | 5.50E-07 | -1.10 | 0.00 | 0.85 | -0.98 | 0.00 | 1.00 |
| Tmem254a | 0.92 | 0.02 | 0.16 | -1.09 | 0.01 | 0.85 | -0.64 | 0.10 | 1.00 |
| Ifit1 | 3.00 | 2.23E-12 | 5.92E-08 | -1.09 | 0.00 | 0.85 | -0.63 | 0.08 | 1.00 |
| Gm22358 | 1.15 | 0.00 | 0.09 | -1.08 | 0.01 | 0.85 | -0.57 | 0.14 | 1.00 |
| Irx2 | 0.33 | 0.39 | 0.53 | -1.03 | 0.01 | 0.85 | -0.85 | 0.03 | 1.00 |
| Ccdc85b | -0.32 | 0.38 | 0.53 | -0.98 | 0.01 | 0.85 | -0.81 | 0.03 | 1.00 |
| Nr2f6 | 0.09 | 0.80 | 0.84 | -0.97 | 0.00 | 0.85 | -1.12 | 0.00 | 1.00 |
| Awat2 | 1.36 | 0.00 | 0.01 | -0.93 | 0.01 | 0.85 | -0.09 | 0.79 | 1.00 |
| Abhd17a | 0.55 | 0.12 | 0.32 | -0.92 | 0.01 | 0.85 | -0.93 | 0.01 | 1.00 |
| Ier5 | 0.45 | 0.18 | 0.39 | -0.90 | 0.01 | 0.85 | -0.97 | 0.01 | 1.00 |
| Znhit2 | 0.05 | 0.86 | 0.89 | -0.89 | 0.00 | 0.85 | -1.00 | 0.00 | 1.00 |
| Socs1 | 0.58 | 0.08 | 0.27 | -0.89 | 0.01 | 0.85 | -0.88 | 0.01 | 1.00 |
| Ifit3 | 2.58 | 1.53E-11 | 1.35E-07 | -0.87 | 0.01 | 0.85 | -0.60 | 0.06 | 1.00 |
| Prpsap1 | 0.35 | 0.22 | 0.43 | -0.87 | 0.00 | 0.85 | -0.66 | 0.02 | 1.00 |
| Fdx1 | 0.26 | 0.38 | 0.53 | -0.86 | 0.01 | 0.85 | -0.95 | 0.00 | 1.00 |
| Hoxa5 | 0.55 | 0.04 | 0.20 | -0.85 | 0.00 | 0.85 | -0.44 | 0.10 | 1.00 |
| Rab5if | 0.52 | 0.06 | 0.23 | -0.82 | 0.00 | 0.85 | -0.65 | 0.02 | 1.00 |
| Usp18 | 1.83 | 6.61E-09 | 1.35E-05 | -0.79 | 0.01 | 0.85 | -0.67 | 0.02 | 1.00 |
| Ifi204 | 1.58 | 1.13E-06 | 0.00 | -0.78 | 0.01 | 0.85 | -0.20 | 0.51 | 1.00 |
| Cmpk2 | 1.21 | 9.11E-06 | 0.00 | -0.77 | 0.00 | 0.85 | -0.61 | 0.02 | 1.00 |
| Ifi44 | 1.99 | 4.37E-10 | 1.29E-06 | -0.77 | 0.01 | 0.85 | -0.86 | 0.00 | 1.00 |
| Cxcl10 | 1.46 | 4.41E-07 | 0.00 | -0.76 | 0.00 | 0.85 | -0.72 | 0.01 | 1.00 |
| Gbp6 | 1.81 | 1.22E-08 | 1.90E-05 | -0.74 | 0.01 | 0.85 | -0.55 | 0.05 | 1.00 |
| Trim30d | 1.56 | 5.62E-08 | 5.52E-05 | -0.73 | 0.01 | 0.85 | -0.32 | 0.22 | 1.00 |
| Ism1 | 0.00 | 0.99 | 0.99 | -0.72 | 0.00 | 0.85 | -0.20 | 0.40 | 1.00 |
| Phlda1 | 0.27 | 0.28 | 0.47 | -0.71 | 0.00 | 0.85 | -0.52 | 0.04 | 1.00 |
| Tefm | 0.58 | 0.02 | 0.16 | -0.68 | 0.01 | 0.85 | -0.14 | 0.57 | 1.00 |
| Cd274 | 1.20 | 4.68E-06 | 0.00 | -0.67 | 0.01 | 0.85 | -0.58 | 0.02 | 1.00 |
| Zmym1 | 0.43 | 0.04 | 0.19 | -0.67 | 0.00 | 0.85 | -0.38 | 0.07 | 1.00 |
| Gm15789 | 0.59 | 0.01 | 0.13 | -0.67 | 0.00 | 0.85 | -0.29 | 0.20 | 1.00 |
| Riox1 | -0.20 | 0.38 | 0.53 | -0.65 | 0.01 | 0.85 | -0.85 | 0.00 | 0.87 |
| Sdf2l1 | 0.09 | 0.63 | 0.71 | -0.65 | 0.00 | 0.85 | -0.61 | 0.00 | 1.00 |
| Gem | 0.48 | 0.03 | 0.18 | -0.64 | 0.00 | 0.85 | 0.20 | 0.37 | 1.00 |
| A4gnt | 0.68 | 0.00 | 0.10 | -0.64 | 0.01 | 0.85 | -0.19 | 0.42 | 1.00 |
| Rab29 | 0.39 | 0.05 | 0.22 | -0.63 | 0.00 | 0.85 | -0.31 | 0.12 | 1.00 |
| Ssbp4 | 0.28 | 0.19 | 0.40 | -0.63 | 0.00 | 0.85 | -0.53 | 0.02 | 1.00 |
| Nectin3 | 0.34 | 0.13 | 0.34 | -0.62 | 0.01 | 0.85 | -0.23 | 0.30 | 1.00 |
| Ccdc88a | 0.65 | 0.00 | 0.09 | -0.61 | 0.01 | 0.85 | -0.30 | 0.17 | 1.00 |
| Med7 | 0.36 | 0.12 | 0.32 | -0.61 | 0.01 | 0.85 | -0.64 | 0.01 | 1.00 |
| Pigx | 0.34 | 0.14 | 0.35 | -0.61 | 0.01 | 0.85 | -0.42 | 0.06 | 1.00 |

**Supplementary Figure S1. Bleomycin-induced gene expression data following treatment with avenciguat or riociguat.** Analysis of down-modulated genes in fibrotic skin of bleomycin-treated mice following avenciguat and riociguat treatment. **(A)** Comparison of gene sets up-regulated by bleomycin (compared with control) and down-regulated by avenciguat or riociguat treatment (compared with bleomycin). **(B)** Top six molecular processes most significantly associated with the 44 genes down-regulated by avenciguat as determined using the Reactome database. **(C)** Expression levels of the same 12 genes per experimental group depicted by z-scores of the averaged and log-transformed expression levels. **(D)** Gene score (singscore) representing the enrichment of the 12 genes in skin from patients with SSc compared with healthy controls. **(E)** Gene score (singscore) representing the enrichment of a generic IFN gene set in skin from patients with SSc compared with healthy controls.


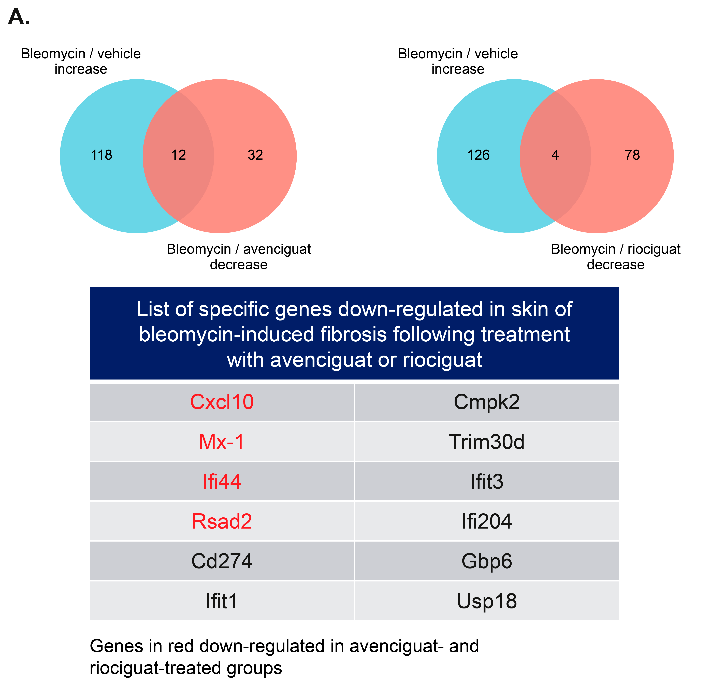

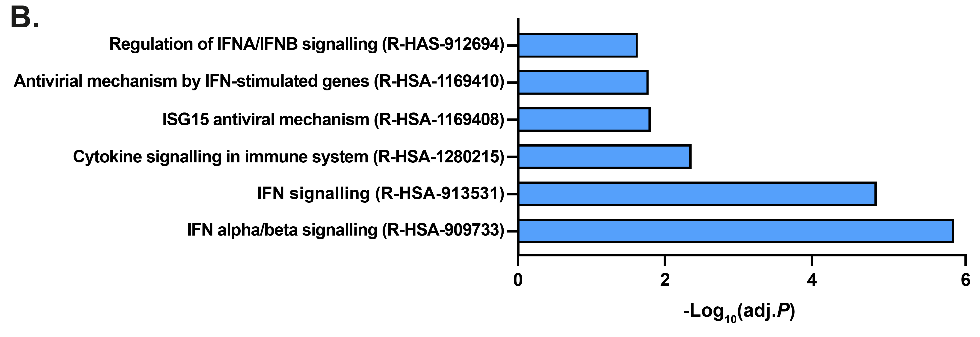

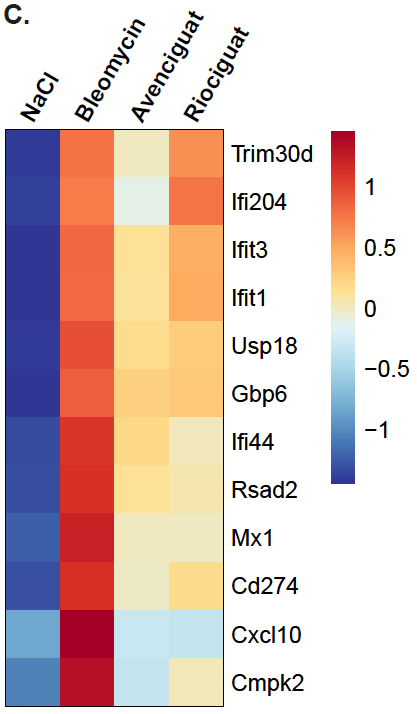


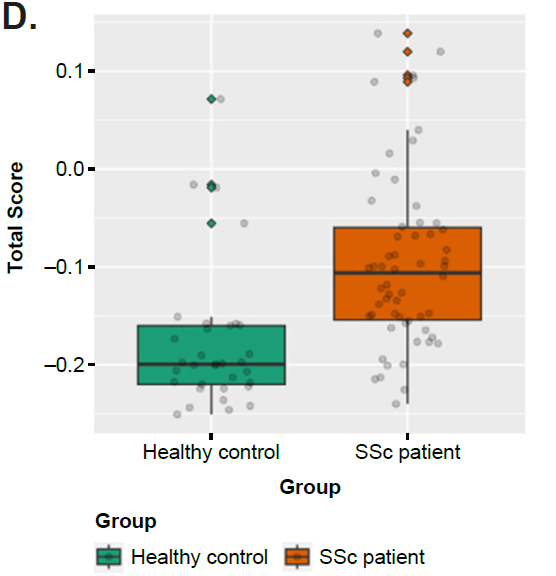

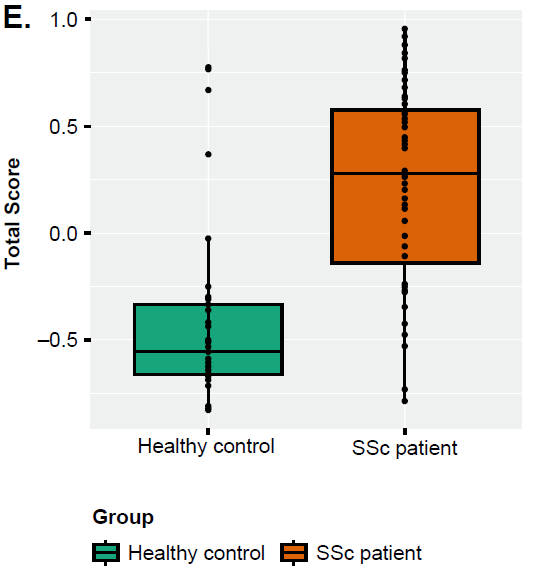


**References**

1. Foroutan M, Bhuva DD, Lyu R, Horan K, Cursons J, Davis MJ. Single sample scoring of molecular phenotypes. *BMC Bioinformatics*. 2018;19:404.

2. Skaug B, Khanna D, Swindell WR *et al.* Global skin gene expression analysis of early diffuse cutaneous systemic sclerosis shows a prominent innate and adaptive inflammatory profile. *Ann Rheum Dis*. 2020;79:379-86.
